# Usefulness of Eye Fixation Assessment for Identifying Type 2 Diabetic Subjects at Risk of Dementia

**DOI:** 10.3390/jcm8010059

**Published:** 2019-01-08

**Authors:** Olga Simó-Servat, Andreea Ciudin, Ángel M. Ortiz-Zúñiga, Cristina Hernández, Rafael Simó

**Affiliations:** 1 Diabetes and Metabolism Research Unit. Vall d’Hebron Research Institute, 08035 Barcelona, Spain; olga.simo@vhir.org (O.S.-S.); aciudin@vhebron.net (A.C.); angelmichaelortizzuniga@gmail.com (A.M.O.-Z.); cristina.hernandez@vhir.org (C.H.); 2 Department of Medicine, Universitat Autònoma de Barcelona, 08193 Barcelona, Spain; 3 Centro de Investigación Biomédica en Red de Diabetes y Enfermedades Metabólicas Asociadas (CIBERDEM), Instituto de Salud Carlos III (ICSIII), 28029 Madrid, Spain

**Keywords:** diabetes, dementia, microperimetry

## Abstract

Type 2 diabetic (T2D) subjects have a significantly higher risk of developing mild cognitive impairment (MCI) and dementia than age-matched non-diabetic individuals. However, the accurate evaluation of cognitive status is based on complex neuropsychological tests, which makes their incorporation into the current standard of care for the T2D population infeasible. Given that the ability to maintain visual gaze on a single location (fixation) is hampered in Alzheimer’s disease (AD), the aim of the present study was: (1) To assess whether the evaluation of gaze fixation during fundus-driven microperimetry correlated with cognitive status in T2D subjects; (2) to examine whether the addition of fixational parameters to the assessment of retinal sensitivity increased the predictive value of retinal microperimetry in identifying T2D subjects with MCI. For this purpose, fixation parameters and retinal sensitivity were compared in three age-matched groups of T2D subjects: normocognitive (n = 34), MCI (n = 33), and AD (n = 33). Our results showed that fixation is significantly more unstable in MCI subjects than normocognitive subjects, and even more altered in those affected by AD (ANOVA; *p* < 0.01). Moreover, adding fixation parameters to retinal sensitivity significantly increases the predictive value in identifying those subjects with MCI: ROC (Receiver Operating Characteristic) Area 0.68 with retinal sensitivity alone vs. ROC Area 0.86 when parameters of fixation are added to retinal sensitivity (*p* < 0.01). In conclusion, our results suggest that fixational eye movement parameters assessed by fundus-microperimetry represent a new tool for identifying T2D subjects at risk of dementia.

## 1. Introduction

The American Diabetes Association recommends individualizing diabetes treatment by taking into account the cognitive capacity of patients [1]. However, the accurate evaluation of cognitive status is based on complex neuropsychological tests [2], which makes their incorporation into current standards of care for the type 2 diabetic (T2D) population unfeasible. This is an important gap that affects clinical practice because T2D patients have a significantly higher risk of developing Alzheimer’s disease (AD) in comparison with age-matched non-diabetic subjects even after adjusting for other risk factors [3,4,5]. These findings have been recently confirmed in a large nationwide population-based study performed in Taiwan with a follow-up of 10 years, which clearly shows that the risk of developing AD in the diabetic population is significantly higher than in the non-diabetic population with a hazard ratio (HR) of 1.7 [6]. Similarly, other longitudinal population-based studies in T2D subjects have shown an association with either an accelerated cognitive decline or an increased incidence of dementia [7]. Because of the increase of diabetes prevalence and the concomitant aging of populations, severe cognitive impairment can be considered as an emerging long-time new “diabetic complication” with dramatic consequences for the affected subjects and their families and with a significant impact on healthcare systems. Mild cognitive impairment (MCI), which is also increased in diabetic patients, consists of cognitive impairment on standard tests but no impairment in activities of daily living and represents a transition state between normal cognitive function and dementia. The annual conversion rate from MCI to dementia ranges between 10–30% in the general population [8,9,10], and we have recently reported that T2D accelerates the conversion to dementia in patients with MCI [11]. Therefore, strategies aimed at identifying T2D patients at risk of dementia are urgently needed. In this regard, it has been estimated that interventions that delay the clinical onset of dementia by 1 year would reduce its prevalence in 2050 by 9 million cases [12].

It could be argued that the benefits of the early diagnosis of dementia are questionable because treatment is still unavailable. However, unrecognized cognitive dysfunction can affect treatment adherence and diabetes self-management resulting in poor glycemic control, an increased frequency of severe hypoglycemic episodes, diabetes-related complications, and hospital admissions [13,14]. In addition, the identification of those T2D subjects affected with MCI would allow the implementation of a more personalized treatment. Hypoglycemic events increase the risk of dementia, therefore treatments that do not increase this risk should be prioritized [15,16,17]. In addition, T2D patients with cognitive impairment would be more prone to present an impaired self-management of diabetes. Thus, the treatment of diabetes should be simplified in these patients in order to increase treatment adherence.

Interestingly, both AD and diabetic complications share common pathogenic pathways. The impairment of insulin signaling, the presence of low-grade inflammation, and the pathways directly related to chronic hyperglycemia, such as the accumulation of advanced glycation end-products (AGEs) and the increase in oxidative stress play an essential role in the pathogenesis of AD [18]. All these pathways have been implicated in diabetic complications and in particular with diabetic retinopathy (DR). Moreover, several pathways involved in neurodegenerative diseases have been identified in human retinas with early stages of DR [19]. These findings indicate that a common etiology exists between the brain and retinal neurodegeneration in the setting of diabetes, which could explain the increased risk of cognitive impairment that T2D subjects present.

Current strategies to screen for dementia are based on neuropsychological tests such as the Mini-Mental State Examination (MMSE), Montreal Cognitive Assessment (MOCA), and the Digit Symbol Substitution Test (DSST) [20]. Both MMSE and MOCA are time-consuming (10 min each), and DSST, which takes only 2 min, has low specificity [20,21]. Additionally, although these tests have an important role as screening tools, they could be influenced by anxiety or depression which occurs in a significant proportion of ageing T2D subjects [13]. Furthermore, when MCI is suspected, a complete neuropsychological battery is needed, which is cumbersome and time consuming (at least 45 min) [22]. Given the high prevalence of T2D in the older population [23], the detection of MCI subjects based on this battery is unfeasible for daily practice.

In clinical practice, there are no reported phenotypic indicators or reliable examinations for identifying T2D patients at risk of developing dementia. In recent years, growing evidence has shown that retinal neurodegeneration is an early event in the pathogenesis of diabetic retinopathy (DR) [24,25]. The retina is ontogenically a brain-derived tissue and it has been suggested that it may provide an easily accessible and non-invasive way of examining the pathology of the brain [25,26]. Therefore, it seems reasonable to propose that the evaluation of retinal parameters related to neurodegeneration could be useful in identifying those T2D patients at a higher risk of developing cognitive impairment and dementia. In this regard, we have recently demonstrated that retinal sensitivity assessed by microperimetry significantly correlated with neuroimaging parameters and cognitive status [27]. Apart from retinal sensitivity, microperimetry permits us to examine the capacity to maintain visual gaze on a single location (fixation), which has been reported as being hampered in AD and other neurodegenerative diseases [28,29,30,31,32].

Abnormalities in oculomotor function and gaze fixation have been previously reported in subjects affected with AD. In fact, several eye movements like pro-saccades, anti-saccades, and the micro-saccades and saccadic intrusions that occur during fixation are altered in subjects with AD [28,29,30]. In addition, during fixation, subjects affected with AD present a greater frequency of saccadic intrusions than normal subjects, causing instability in gaze maintenance [29]. Moreover, micro-saccades that occur during normal fixation in AD are notably more oblique compared with normal adults [30]. However, little is known regarding the potential usefulness of the assessment of gaze fixation for identifying T2D patients at risk of AD. This is an important issue because the neural circuits involved in gaze fixation are not the same as those participating in retinal sensitivity and, therefore, complementary information could be obtained.

On this basis, we hypothesize that the assessment of retinal fixation by means of fundus-driven microperimetry could be useful to identify those diabetic subjects with cognitive impairment. For the first time we have analyzed data regarding fixational eye movement parameters assessed by microperimetry with the following aims: (1) To assess whether gaze fixation abnormalities in T2D diabetic subjects are related to cognitive status and, therefore, could be used as a biomarker for identifying T2D subjects at risk of dementia, and (2) to determine whether fixation study is able to increase the diagnostic reliability of retinal sensitivity in identifying subjects at risk of cognitive impairment. Since in diabetic subjects with advanced diabetic retinopathy, both sensitivity and fixation could be altered [33], those subjects with moderate non-proliferative or more advanced stages of diabetic retinopathy were excluded from the study.

## 2. Materials and Methods

Data regarding fixation parameters obtained from the nested case-control study DIALRET (ClinicalTrial.gov: NCT02360527) were analyzed. A total of 33 patients with AD, 33 with MCI, and 34 with normal cognition in whom a full report of fixation parameters was available were included in the study. All these patients were selected from 214 consecutive type 2 diabetic patients attending a memory clinic (Fundació ACE, Barcelona, Spain). The study was conducted according to the Declaration of Helsinki and was approved by the local Ethics Committee.

The main inclusion criteria were: (a) Age >65 years; (b) type 2 diabetes with a duration >5 years; (c) written informed consent which included accepting participation in brain MRI (Magnetic Resonance Imaging) and PET (Positron Emission Tomography), as well as a potential lumbar puncture.

The main exclusion criteria were: (a) Patients with other neurodegenerative diseases of the brain or retina (i.e., glaucoma) or cerebrovascular diseases (Fazekas scale score ≤1) [34]; (b) HbA1c >10% (86 mmol/mol). The exclusion of patients with poor control was because very high blood glucose levels could affect retinal function [35]. (c) Moderate non-proliferative diabetic retinopathy (DR) or more advanced stages of DR according to the International Clinical Diabetic Retinopathy Disease Severity Scale [36]. The exclusion of patients with more advanced DR was based on the fact that severe microvascular impairment could participate in neurodegeneration, and the aim of the study is to assess retinal neurodegeneration regardless of the presence of overt microangiopathy.

All patients underwent complete neuropsychological, neurological, and psychiatric evaluations as previously described [22] including Mini-Mental State Examination (MMSE) and Alzheimer’s Disease Assessment Scale-Cognition subscale (ADAS-Cog). Higher values on MMSE and lower values on ADAS-Cog evaluations indicate better cognition. A neuropsychologist and a neurologist from a memory clinic performed the cognitive assessment and the diagnosis of MCI and AD. All patients diagnosed with MCI fulfilled MCI Petersen’s diagnosis criteria [8,37]. In addition, a biochemical analysis including HbA1c and a lipid profile was performed.

Retinal sensitivity was evaluated by fundus driven microperimetry (MAIA 3rd generation, Centervue^®^) after a previous papillary dilation of minimum 4mm. The standard MAIA test covers 10° diameter area with 37 measurement points and a red 1° radius circle was used as the fixation target.

**Figure 1:**
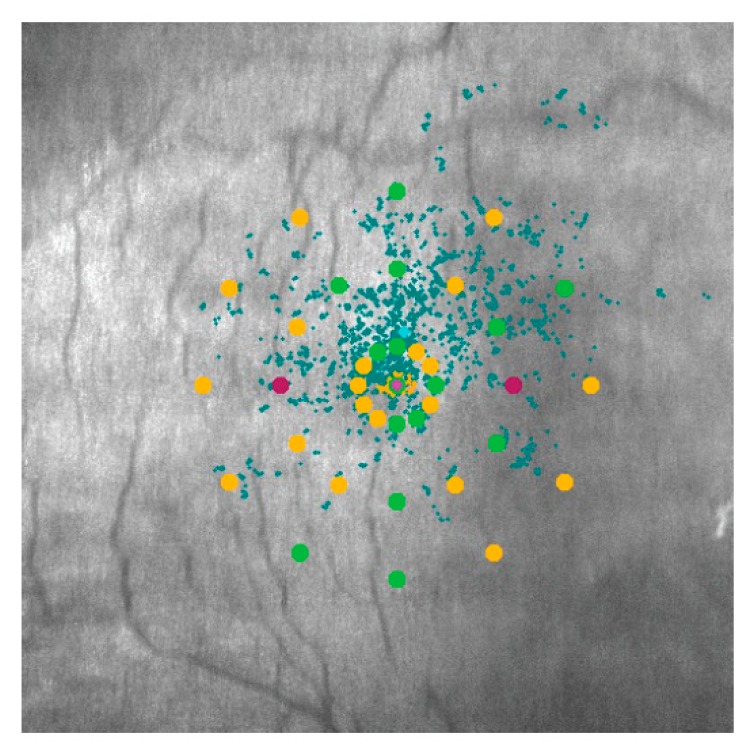
Microperimetry corresponding to a mild cognitive impairment (MCI) subject. The circles in green, yellow, and purple are the 37 measurement points in which retinal sensitivity is assessed. Different colors indicate different retinal sensitivity at each point (green being the best and purple the worst). The blue dots are the fixation points detected during the whole examination (3.5 min).

For the evaluation of fixation, the MAIA microperimeter uses high-speed eye trackers (25 Hz). The parameters that evaluate fixation are assessed by tracking eye movements 25 times/Sec and by plotting the resulting distribution over the SLO (Scanning Laser Ophthalmoscope) image. Each movement is represented by a point in the distribution which will constitute the fixation pattern (Figure 1). All fixation positions during the examination are used by the instrument to calculate the fixation indexes P1 and P2, which represent the percentage of fixation points inside a circle of 2 and 4 degrees of diameter from the total of fixation points, respectively. Subjects were classified as having stable fixation if P1 was more than 75%, relatively unstable fixation if P1 was less than 75% and P2 more than 75%, and unstable fixation if both P1 and P2 were below 75%. Microperimetry also provides a more accurate estimation of the fixation pattern using the bivariate contour ellipse area (BCEA). It calculates, by using two orthogonal diameters that describe the extent of the fixation points (in degrees), the area and orientation of an ellipse encompassing a given proportion of the fixation points. Lower BCEA values define better fixation stability. We have selected the BCEA corresponding to 95% of the fixation points [38,39]. For retinal sensitivity and fixation, data corresponding to the right eye were used, which was the first eye explored in all subjects. The duration of the measurements varies from 1 min to 4 min approximately, depending on the subject.

The reliability index is assessed by measuring the number of stimuli reported as seen by the patient when a stimulus is projected onto the optic nerve head (blind spot). A 100% of reliability means that none of the stimulus projected onto the nerve head is reported as seen [39].

### Statistical Analysis

The categorical variables are presented as percentages. For the quantitative variables, the mean and standard deviation (within the gaps) is displayed, except for BCEA95 in which the median and range are used. Statistical significance was accepted at *p* < 0.05. To assess differences between groups, the Chi-square test for qualitative variables and ANOVA followed by LSD (Least Significant Difference) post-hoc tests for quantitative variables were used. For BCEA95, which does not have a normal distribution (assessed by Shapiro Wilk test, cut-off *p* < 0.05), a non-parametric test was used to compare between groups (Kruskal-Wallis). For the significant differences observed, post hoc pairwise comparison analysis using the Duncan method was performed.

To evaluate the correlation between BCEA95 and ADAS-Cog or MMSE, Spearman’s correlation test and regression analysis were performed. Significance was accepted at the level of *p* < 0.05. The Bonferroni correction was used for multiple comparisons.

Logistic regression analysis to predict MCI vs. normocognitive status was performed by using the following variables: Age, sex, retinal sensitivity, fixation indexes P1 and P2, and BCEA95. All possible equations were analysed and the two models that presented a highest AUC (Area Under the Curve) were the following: (1) Retinal sensitivity + P1; (2) retinal sensitivity + P1 + BCEA95. Since the purpose of the study was to evaluate fixation parameters, and as BCEA95 was one of the best fixation measurements, we selected the second model. Multicollinearity was assessed by calculating the Variance Inflation Factor (VIF). ROC curves and the Chi-squared test for ROC area comparison were performed. Statistical analyses were performed with the Stata statistical package.

## 3. Results

The main clinical characteristics of subjects with type 2 diabetes included in the study are summarized in Table 1. No differences were found among groups in terms of age, sex, hypertension, dyslipidaemia, BMI (Body Mass Index), diabetes duration, or HbA1c. The mean reliability index of the microperimetry test was above 90% in all groups and as expected, retinal sensitivity decreased in the group with MCI and especially in those with AD (*p* < 0.01). The time taken for the examination was longer in AD subjects or MCI (*p* < 0.01). This is because when retinal sensitivity is hampered, the examination takes a longer time since more stimuli and different intensities are presented to the patient. On the other hand, when retinal sensitivity is preserved, only the stimuli with the lowest intensity are presented and the examination takes less time.

**Figure 2:**
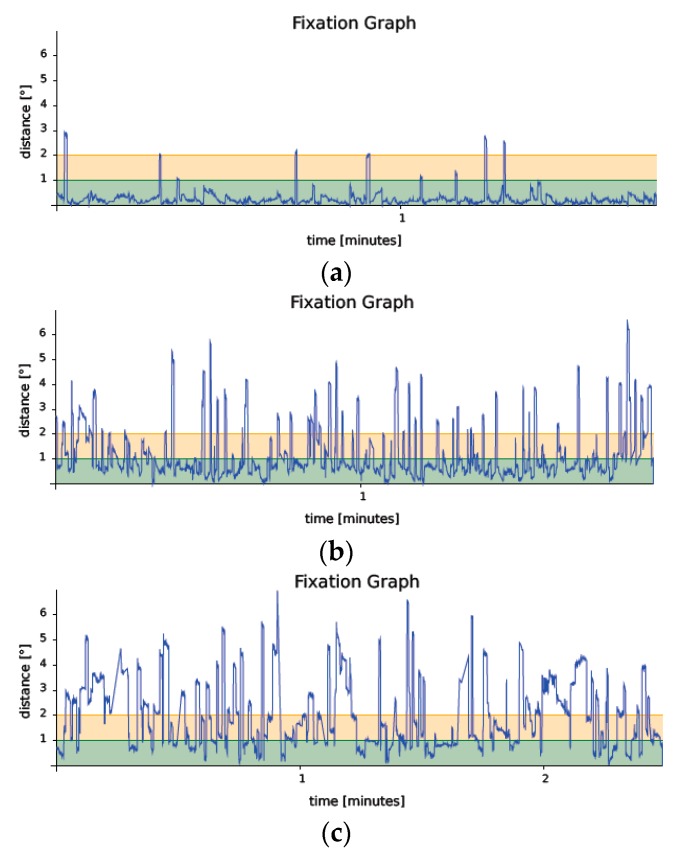
Representative examples of the fixation graph close to the median BCEA95 value of a normocognitive (**a**) (BCEA95 3.1°), MCI (**b**) (BCEA95 34.7°) and AD (**c**) (BCEA95 40.4°) subject. The green and the orange bands represent a circle of 1 and 2 degrees respectively.

Fixation was more unstable, in terms of P1 and P2 percentages, in the group with MCI and especially in the group with AD. Therefore, the lowest fixation capacity was found in patients with AD and the highest in patients with normocognition, the differences between the groups being statistically significant (*p* < 0.00001). To illustrate this point, representative examples of the fixation graph, which describes the amplitude of eye movements in degrees versus time, of each group (normocognition, MCI, and AD) are shown in Figure 2. In addition, we also found a significant difference among groups BCEA95 (*p* < 0.00001). In the pairwise comparison analysis, the differences were statistically significant among the three groups as shown in Table 2.

**Figure 3:**
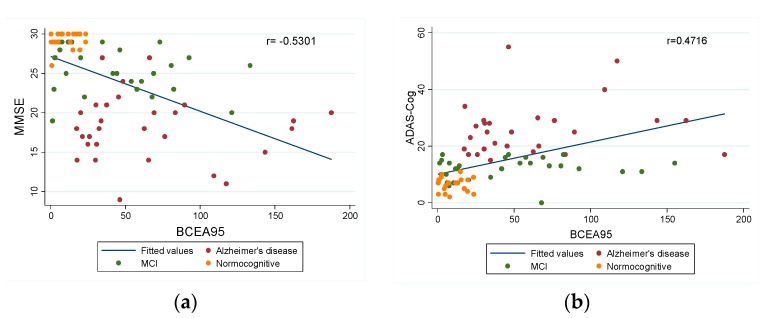
Linear regression graphs between: (**a**) Mini-mental State Examination (MMSE) and BCEA95; (**b**) Alzheimer’s Disease Assessment Scale-Cognition subscale (ADAS-Cog) and BCEA95.

A significant negative correlation was found between BCEA95 and MMSE (r = −0.5301, *p* < 0.00001). In addition, BCEA95 also significantly correlated with the ADAS-Cog (r = 0.4716, *p* < 0.00001). In other words, a more unstable fixation correlated with lower punctuation in the MMSE test and a higher score in the ADAS-Cog, indicating that fixation instability is associated with a degree of cognitive impairment (Figure 3a,b).

Retinal sensitivity, BCEA95, P1, and P2 were also correlated among them, and the coefficients (Spearman correlation) and their significance are displayed in Table 3. Interestingly, when analyzing the same correlation within the normocognitive and MCI groups (excluding those with AD), fixation P1 and P2 values no longer correlate with retinal sensitivity (Pearson correlation coefficients: 0.3199 and 0.311 respectively, *p* value > 0.05).

**Figure 4:**
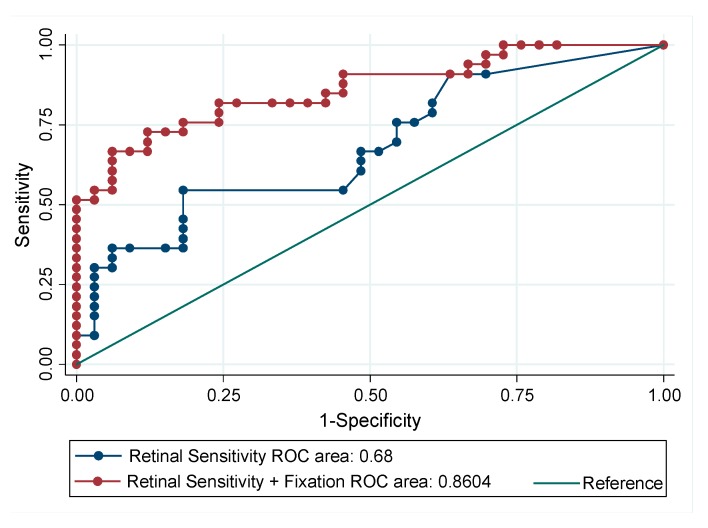
Comparison of two prediction models for MCI versus normal cognition, one based on retinal sensitivity and fixation parameters P1 and BCEA95, and one with retinal sensitivity alone.

Finally, a comparison regarding the capacity to discriminate MCI subjects from normocognitive subjects by using retinal sensitivity alone or by adding to the predictive model the parameters of fixation BCEA95 and P1 was performed. The results are summarized in Table 4 and Figure 4. The addition of fixation assessment to the results obtained by measuring retinal sensitivity significantly increased the ROC area (*p* = 0.01), thus enhancing the capacity to discriminate between normocognitive and MCI diabetic subjects. The optimal cut-off based for maximum efficiency (the cut-off point that maximizes the percentage of correct classifications) was 0.494191 with a sensitivity of 72.7% (95% CI: 55.8 to 84.9%), specificity of 87.9% (95% CI: 72.7 to 95.2%), positive predictive value of 85.7% and a negative predictive value of 76.3%. The VIF values (<10) showed that multicollinearity was not present among the predictors (Table 5).

## 4. Discussion

Our findings indicate that fixational eye movement parameters assessed by microperimetry are useful for identifying T2D subjects with MCI. Moreover, the addition retinal fixation parameters, and more specifically P1 and BCEA95, to the assessment of retinal sensitivity significantly increase the capability to identify those subjects with MCI. Therefore, the retinal functional assessment of ocular movements as an objective method for identifying T2D patients with MCI opens up new feasible clinical management procedures addressed to testing cognitive status, which could easily be added to the routine ophthalmologic examination for the screening of DR.

Although there is evidence indicating that ocular movements are altered in AD, little is known about the usefulness of the study of ocular movements to identify the prodromal stages of dementia. Kapoula et al. [30] observed that MCI subjects present a major prevalence of oblique micro-saccades during fixation compared with control subjects. Lagun et al. [40] demonstrated that the incorporation of ocular movement information (i.e., duration of fixation, saccade length, and direction) was able to increase the specificity and sensitivity of the measurements of the visual-paired comparison task previously reported. Techniques used to evaluate ocular movements were based on electrodes placed in the region around the eyes and usually incorporated support to restrict head movements [29,41]. New eye-tracking systems have been developed and are usually video-based techniques, which has the advantage of being non-invasive [41,42,43]. One of their main limitations is head movement, because even small movements may cause large errors in the estimation of a calibrated tracker [42]. Fundus-driven microperimetry is a video-based technique with the advantage of incorporating a chin and forehead bars that restrict head movements. However, the exact distance between the optical stimuli and the eye could vary between subjects due to anatomical reasons and this could represent a limitation when retinal fixation is being assessed.

As previously mentioned, the presence of advanced DR could hamper the microperimetry results. In this subset of subjects, the screening of cognitive impairment should be based on other methods not based on retinal assessment (i.e., neuropsychological tests). However, it should be noted that the vast majority of T2D subjects present no DR or just mild non-proliferative DR. In the present study, given that the T2D patients included did not present signs of DR or other retinal disease, the alterations in fixation detected in those patients with cognitive impairment could be attributed to any neurodegenerative disease rather than an intrinsic eye disease. Marseglia et al. [44] showed that the presence of T2D affects perceptual speed, attention, and primary memory in patients of 70–75 years. They postulate that these domains might be primarily affected by diabetes. It should be noted that fixation eye movements are affected by attention and working memory [28]. For example, micro-saccade rates transiently decrease during an attentional task [45]. Therefore, the evaluation of fixation by fundus-driven microperimetry could be a biomarker of the first neuropsychological abnormalities that occur in diabetes-related cognitive decline. The omnipause neurons (located in the nucleus raphe interpositus of the paramedian pontine reticular formation) or the superior colliculus (SC) are two of the main brain areas involved in fixation. It is possible that these areas are affected early in the neurodegenerative process [46]. During microperimetry, the subjects are asked to maintain their gaze fixated on a central target, while stimuli with different intensities are presented at 37 points in 3 concentric circles of 2, 6, and 10 degrees of diameter [47]. It could be hypothesized that subjects with cognitive impairment are unable to inhibit the saccades triggered by the most eccentric stimuli and maintain their gaze fixated on a central target. Furthermore, certain areas of the cerebral cortex, such as the parietal and frontal cortex (frontal eye fields and the dorsomedial prefrontal cortex), show elevated firing rates during fixation. These areas could contribute to the control of fixation through descending projections to the SC and the omnipause region [46].

We observed statistically significant differences in P1, P2, and BCEA95 among the three groups. Moreover, a correlation was found between BCEA95 and either ADAS-Cog or MMSE punctuations. In addition, when BCEA95 and P1 were added to retinal sensitivity assessment, the capacity to detect MCI by using microperimetry increased significantly. In fact, for a cut-off point of 0.49 of the model that combines retinal sensitivity and fixation parameters (P1 and BCEA95), microperimetry has a sensitivity of 72.7% and a specificity of 87.9% in identifying the subjects with MCI. Furthermore, we did not find collinearity between parameters of retinal sensitivity and fixation. This would mean that this combined test is examining different neural circuits, thus improving the performance of the examination. In this regard, retinal sensitivity relies on the retina and the neural pathway of vision. The first station of the optic tract is the lateral geniculate body of the thalamus, which is the first major visual processing region in the brain and plays a crucial role in relaying information from the retinal ganglion cells to the primary visual cortex [48]. By contrast, the superior colliculus and the parietal and frontal cortex play an essential role in gaze fixation. There is evidence that both the geniculate body and the superior colliculus are affected by AD [49,50,51]. Therefore, gaze fixation and retinal sensitivity are measuring different neurological pathways and, consequently, can be considered complementary measurements of neurodegeneration, thus enhancing the reliability of the retinal sensitivity assessment alone.

The main limitation of our study is that we have included patients without DR or only those patients with mild non-proliferative DR. Since the presence of more advanced stages of DR, and in particular diabetic macular edema, can alter fixation [52], our results are not representative for the whole diabetic population. Furthermore, longitudinal studies examining retinal fixation in diabetic subjects with and without DR should be performed in order to fully address the utility of fixation assessment by microperimetry and guide its appropriate use.

In conclusion, our results indicate that the assessment of gaze fixation abnormalities in T2D is related to cognitive status and could be a useful tool for identifying patients at risk of developing dementia. In addition, the assessment of retinal sensitivity in combination with parameters of fixation by using microperimetry could be a reliable method for detecting prodromal stages of dementia in the T2D population.

## Figures and Tables

**Table 1:** General characteristics and parameters of retinal sensitivity and fixation.

	AD *n* = 33	MCI *n* = 33	Normocognitive *n* = 34	*p*
Age (years)	79 (5.57)	77.03 (4.38)	75.53 (6.97)	0.06
Gender (males, %)	42.42	51.52	58.82	0.41
BMI	27.72 (3.79)	28.28 (4.48)	30.28 (4.45)	0.61
Hypertension (%)	78.79	75.76	73.53	0.88
Dyslipidemia (%)	63.64	66.67	71.43	0.81
Diabetes duration (years)	10.45 (6.23)	13.73 (10.64)	10.91 (6.12)	0.22
HbA1c (% of Hb, DCCT *)	6.79 (1.02)	6.99 (1.25)	7.5 (0.95)	0.37
Retinal sensitivity (dB)	16.95 (6.01)	21.05 (4.82)	23.39 (2.47)	<0.00001
Fixation P1 (%)	45.03 (24.89)	58.21 (26.66)	88.52 (12.19)	<0.00001
Fixation P2 (%)	70.2 (20.61)	81.03 (15.60)	96.09 (3.66)	<0.00001
BCEA95 (degrees) (median, range)	46.3 (12.5–187.7)	34.4 (1.2–155.1)	4.55 (0.3–23.5)	<0.00001
Reliability	92.83 (14.49)	97.45 (7.04)	96.59 (13.59)	0.28
Duration of the examination (minutes)	3.1 (1.14)	2.34 (0.83)	1.71 (0.42)	<0.00001

* DCCT: Diabetes Control and Complications Trial was the reference method to calculate the HbA1c.

**Table 2:** Pairwise comparison analysis (contrast and 95% confidence interval).

	Normocognitive vs. MCI	MCI vs. AD	Normocognitive vs. AD
Retinal Sensitivity	4.10 (1.78, 6.41)	2.33 (0.08, 4.59)	6.43 (4.00, 8.87)
Fixation P1	13.18 (2.10, 24.26)	30.30 (19.49, 41.11)	43.48 (31.83, 55.14)
Fixation P2	10.83 (3.39, 18.27)	15.06 (7.80, 22.32)	25.89 (18.06, 33.72)
BCEA95	−23.27 (−41.23, −5.31)	−35.11 (−52.93, −17.28)	−58.37 (−77.13, −39.61)

**Table 3:** Spearman correlation coefficients (and *p* values*) between retinal sensitivity and fixation parameters.

	Fixation P1	Fixation P2	BCEA95
Retinal Sensitivity	0.41 * <0.001	0.46 * <0.00001	−0.47 * <0.00001
Fixation P1	-	0.93 * <0.00001	−0.93 * <0.00001
Fixation P2	-	-	−0.96 * <0.00001

**Table 4:** ROC Area comparison between retinal sensitivity alone, and retinal sensitivity and fixation parameters (P1 and BCEA95). The outcome is mild cognitive impairment (MCI) (reference = normal cognition).

	ROC Area	Std. Err.	95% CI	*p*-Value
Retinal Sensitivity	0.68	0.07	0.55–0.81	<0.01
Retinal Sensitivity + BCEA95 + Fixation P1	0.86	0.05	0.77–0.95	

**Table 5:** Multicollinearity assessment: Variance inflation factor (VIF) for each predictor.

	VIF	1/VIF
Retinal Sensitivity	1.15	0.87
BCEA95	4.45	0.22
Fixation P1	4.32	0.23
